# Decision tree model predicts live birth after surgery for moderate-to-severe intrauterine adhesions

**DOI:** 10.1186/s12884-022-04375-x

**Published:** 2022-01-29

**Authors:** Ru Zhu, Hua Duan, Wenbin Xu, Sha Wang, Lu Gan, Qian Xu, Jinjiao Li

**Affiliations:** 1 Department of Minimally Invasive Gynecology, Beijing Obstetrics and Gynecology Hospital, Capital Medical University, Beijing, 100006 China; 2 Department of Obstetrics and Gynecology, Anqing Hospital Affiliated to Anhui Medical University, Anqing, 246003 China; 3 Department of Medical Genetics, Institute of Basic Medical Science, Chinese Academy of Medical Science & Peking Union Medical Collage, Beijing, 100005 China

**Keywords:** Hysteroscopic adhesiolysis, Pregnancy, Intrauterine adhesion

## Abstract

**Background:**

After treatment of intrauterine adhesions, the rate of re-adhesion is high and the pregnancy outcome unpredictable and unsatisfactory. This study established and verified a decision tree predictive model of live birth in patients after surgery for moderate-to-severe intrauterine adhesions (IUAs).

**Methods:**

A retrospective observational study initially comprised 394 patients with moderate-to-severe IUAs diagnosed via hysteroscopy. The patients underwent hysteroscopic adhesiolysis from January 2013 to January 2017, in a university-affiliated hospital. Follow-ups to determine the rate of live birth were conducted by telephone for at least the first postoperative year. A classification and regression tree algorithm was applied to establish a decision tree model of live birth after surgery.

**Results:**

Within the final population of 374 patients, the total live birth rate after treatment was 29.7%. The accuracy of the model was 83.8%, and the area under the receiver operating characteristic curve (AUC) was 0.870 (95% CI 7.699–0.989). The root node variable was postoperative menstrual pattern. The predictive accuracy of the multivariate logistic regression model was 70.3%, and the AUC was 0.835 (95% CI 0.667–0.962).

**Conclusions:**

The decision tree predictive model is useful for predicting live birth after surgery for IUAs; postoperative menstrual pattern is a key factor in the model. This model will help clinicians make appropriate clinical decisions during patient consultations.

## Background

Intrauterine (IU) adhesions (IUAs) result from injury to the basement layer of the endometrium. Various kinds of damage can cause adhesion between the walls of the uterus with accompanying adverse morphological changes, or loss of function of the uterine cavity, or both [1]. IUAs are mainly indicated by abnormal menstruation (oligomenorrhea or amenorrhea) and fertility disorders (e.g., infertility, repeated pregnancy loss, premature delivery and obstetric complications) [2]. The rate of IUAs after curettage has been reported at 19.1%, of which 42% were moderate-to-severe [1]. The results of transcervical resection of adhesions (TCRA) have not been satisfactory; recurrence of serious IUAs is 62.5%, and the pregnancy rate is only 25% [3].

IUAs seriously harm women’s reproductive, physical, and mental health. For patients with IUAs as the main cause of infertility, the degree of adhesions is closely related to the outcome of pregnancy [4]. The factors that affect live birth after surgery include age [5], menstrual pattern, endometrial thickness, times of separation of IUAs, the cause of IUAs, and the location of adhesions [6–11]. The pregnancy outcome of IUAs is uncertain, which greatly complicates doctors’ clinical decision-making and patient consultations. The lack of preoperative evaluation is likely to increase the risk of ineffective and excessive treatment.

A multivariate logistic regression analysis model can predict the clinical outcome and identify risk factors, but is difficult to implement and explain, especially for clinicians without a statistical background [12]. It is difficult to use and promote in clinical work. The results are displayed as a tree structure, which is greatly convenient for clinicians [12–17].

The main purpose of treatment for patients with IUAs is to obtain live births. To better evaluate and predict live birth after surgery for IUAs and provide clinical consultation [17], the present study reviewed retrospective data to build and verify a decision tree model.

## Material and methods

### Patients

Patients with moderate or severe IUAs were selected who had been treated for infertility or recurrent abortion, from January 2013 to January 2017, at the Gynecology Minimally Invasive Center of Beijing Maternity Hospital Affiliated to Capital Medical University in China. This is a tertiary medical institution and hysteroscopic diagnosis and treatment center with nearly 1000 hysteroscopic surgeries performed annually.

For inclusion, each patient was aged 20 to 40 years; with an outpatient hysteroscopic diagnosis of moderate or severe IUAs (AFS ≥ 5) [18]; secondary infertility; and ovulation by ultrasound. Patients with any of the following were excluded: the shape of the uterine cavity did not recover; no second look was performed ≤3 months after the operation; there were uterine malformations, endometrial lesions, or tuberculosis of the reproductive system; serious adenomyosis; or abnormal semen of the male partner (World Health Organization version 5 standard).

### Hysteroscopic adhesiolysis

The cervix was softened by 400 μg of misoprostol, 12 h before the operation. Tracheal intubation and intravenous general anesthesia were administered. Olympus S70 surgical hysteroscopy series equipment was used, and the perfusion medium was normal saline. Under the direct view of the hysteroscope, the shape of uterine cavity, and the position and degree of adhesion were observed. Adhesion tissue was separated via needle electrode and scar tissue by ring electrode, expanding the uterine cavity volume and removing scar tissue with care to protect the residual endometrium [19]. Successful separation of IUAs was defined as restoration of normal IU anatomy, display of bilateral uterine angle, no adhesions [20].

The operator was a senior doctor with a surgical team and extensive experience in the separation of IUAs. A physical barrier (intrauterine suitable balloon or Foley balloon or heart-shaped copper intrauterine device) was used to prevent the recurrence of IUAs [21]. All patients were given postoperative routine prevention of infection and hormone cycle therapy to promote endometrial growth [21]. Hormone therapy was started on the second day after the operation and consisted of estradiol valerate at 4 mg/d for 21 days, with the addition of dydrogesterone at 20 mg/d for the final 10 days of estrogen therapy. This was followed by a period of 7 days of no hormone therapy. The hormone therapy regimen was then repeated for an additional 2 months.

### Research variables

Live birth is defined as delivery occurring at any time between 28 and 42 completed weeks of gestation. In this study, the diagnosis and score of IUAs referred to the American Fertility Society (AFS) standard of 1988. According to the nature, scope, and menstrual pattern of adhesions, the quantitative scores were: mild, 1–4 points; moderate, 5–8 points; or severe, 9–12 points.

For measurement of uterine volume and endometrial thickness, all patients were examined by vaginal color ultrasound (model: GE E8). Patients with menstruation were examined during the latter period of menstruation proliferation. Patients with amenorrhea were examined at no specific time. The length, width, and thickness of the uterine body were measured. The length was considered the distance from the bottom of the uterus to the internal orifice of cervix. The width and thickness of the uterine body were the transverse diameters of the coronal and sagittal sections, respectively. The volume of the uterus was calculated as length × width × thickness × 0.523, in cm3 [22]. The depth of the uterine cavity is the distance from the bottom of the endometrial cavity to the external orifice of the cervix.

According to the modified vas method of Osada et al. [23], the patients’ previous menstruation was judged as either normal or amenorrhea. For those with normal previous menstruation, the changes in menstrual volume before and after surgery were compared. There were three types of postoperative menstrual patterns: normal menstrual volume, improvement of menstruation, and no improvement of menstruation. The causes of IUAs were divided into three categories: early pregnancy termination; middle and late pregnancy termination; or non-pregnancy related factors.

### Follow-up observations

Data was collected from the electronic database and telephone return visits of inpatients, including demographic and clinical characteristics, surgical records and hysteroscopic pictures, pregnancy outcome, and related complications after treatment. The pregnancy outcome was followed up by telephone in February 2018.

Three months after the operation, menstruation was evaluated for improvement in the outpatient department, and hysteroscopy was performed [20]. A normal anatomy of the uterine cavity and no cause of infertility outside the uterine cavity suggested that natural conception was possible. If there were other infertility factors or natural pregnancy failure, patients were recommended to receive assisted reproductive technology.

### Obstetric delivery mode and obstetric complications

A clinical pregnancy was defined as within the IU gestational sac. Live birth was ≥28 weeks of gestation, with a live birth obtained. Full term birth was considered ≥37 weeks of gestation, and premature birth < 37 weeks of gestation. Loss of pregnancy was recorded as spontaneous abortion, fetal arrest, or stillbirth.

### Statistical analysis

The final analysis comprised 374 patients (i.e., samples), including 23 variables, with live birth as the outcome variable and 22 other related factors as the predictive variables (Table 1). Because the positive and negative samples of the original data were not uniform (live production = 111, inactive production = 263, total sample size = 374), we used the ROSE (Randomly Over Sampling Examples) package in R language [24, 25] to deal with class imbalance. We under-sampled multiple data and oversampled less data, while keeping the new sample size the same as the original sample size. Moreover, we set the parameter *p* = 0.5 of the resample function in ROSE to keep the proportion of positive sample size approximatively equal to 0.5. Consequently, the number of samples with live and non-live production is more or less balanced (live production = 173, non-live production = 201, total sample size 374). The new data was used for further analyses.

**Table 1:** Attribute variables or categories of each influencing factor

	Data type	Code
Live birth^a^	2 categories	No = no live birth, yes = live birth
Age	Continuity	
Times of pregnancy	Frequency	
Childbirth	2 categories	No = no, yes = yes
Chief complaint	3 categories	a = primary infertility, b = secondary infertility, c = recurrent abortion
Menstrual pattern	3 categories	a = less than 1/4, b = 1/4 ~ 1/2, c = more than 1/2
Etiology	3 categories	a = primary infertility, b = secondary infertility, c = recurrent pregnancy
Pregnancies loss	3 categories	*z* = 0 times, a = 1 time, b = more than or equal to 2 times
IU operations	Frequency	
History of TCRA	2 categories	No = yes, yes = no
Endometrial thickness	3 categories	a = ≤3 mm, b = 4–6 mm, c = ≥7 mm
Uterine volume	Frequency	
Preoperative AFS	Frequency	
Degree of adhesion	2 categories	a = moderate, b = severe
Adhesion type	2 categories	a = mixed type, b = peripheral type
IU depth preop, mm	Frequency	
Uterine horn closure preop, *n*	3 categories	*z* = 0 side, a = 1 side, b = 2 side
Tubal ostia preop	3 categories	*z* = 0 side, a = 1 side, b = 2 side
Isolation barrier	3 categories	a = balloon, b = uterine suitable balloon, c = IUD
Postoperative AFS	Frequency	
Tubal ostia postoperative	3 categories	*z* = 0 side, a = 1 side, b = 2 side
Menstrual patterns postoperative	3 categories	*z* = improvement, a = no improvement, b = normal amount
IU depth postoperative	Continuity	

*Preop* preoperative, *TCRA* transcervical resection of adhesions

^a^ The only dependent variable; the remaining are independent

The R language (www.r-project. ORG) random Forest package was used to rank the features of the independent variables (predictive variables) according to the mean decrease accuracy index. The first 10 most important feature variables were selected. To get the best predictive efficiency, various data partition schemes were tried. We finally choose 90% of the original data to build the decision tree model, a classification and regression tree (CART) algorithm, and 10% of the data to verify the model. The decision tree model was constructed using the R language rpart (Recursive Partitioning and Regression Trees) software package. Using the rpart package, the decision tree was constructed by CART algorithm based on gini index splitting criteria. The minimum sample size of the parent node was 30, the minimum sample size of the leaf node was 10, and the depth was set to 5.

The logistic regression model was constructed with the top 10 variables using the enter method (SPSS 23.0). *P* < 0.05 was statistically significant.

## Results

### Clinical outcome

From January 2013 to January 2017, 394 women were treated for moderate and severe IUAs. Of these women, 12 were lost to follow-up, and there were 4 excluded for serious adenomyosis. In addition, 2 women each had endometrial lesions or were not completely restored to normal IU morphology after surgery.

Thus, the study population comprised 374 patients with complete data. The average age was 31.5 ± 4.09 (20–40) years, the follow-up time was 24.1 ± 3.2 (12–58) months, and the average time from surgery to pregnancy was 7.4 ± 5.4 (1–25) months. The patients included in the study had no serious surgical complications. The overall population postoperative rates for pregnancy, live birth, and pregnancy loss were, respectively, 40.6% (152/374), 29.7% (111/374), and 10.9% (41/374). For the group with severe IUAs, rates of pregnancy and live birth were 33.6% (44/131) and 22.1% (29/131). Women with moderate IUAs showed corresponding rates of 44.4% (108/243) and 33.7% (82/243). Factors related to live birth are shown in Table 2.

**Table 2:** Factors affecting live birth after hysteroscopic adhesiolysis

		No	Yes	*P*
Subjects, *n*		263	111	–
Age, y		31.46 ± 4.30	31.35 ± 3.77	0.811
Times of pregnancy		2 (1–3)	2 (1–3)	0.204
Chief complaint	Primary infertility	16 (6.1%)	3 (2.7%)	0.272
	Secondary infertility	209 (79.5%)	88 (79.3%)	
	Recurrent pregnancy loss	38 (14.4%)	20 (18.0%)	
Childbirth	No	216 (82.1%)	94 (84.7%)	0.653
	Yes	47 (17.9%)	17 (15.3%)	
Menstrual pattern		0.33 (0.20–0.50)	0.50 (0.33–0.50)	0.009*
Etiology	Non-pregnancy-related	23 (8.7%)	4 (3.6%)	0.152
	Termination early pregnancy	201 (76.4%)	92 (82.9%)	
	Termination mid-late pregnancy	39 (14.8%)	15 (13.5%)	
Pregnancies lost, *n*	0	164 (62.4%)	55 (49.5%)	0.033*
	1	61 (23.2%)	36 (32.4%)	
	≥2	38 (14.4%)	20 (18.0%)	
IU operations, *n*		0 (0–0)	0 (0–0)	0.400
History of TCRA, *n*	0	211 (80.2%)	101 (91.0%)	0.011*
	≥1	52 (19.8%)	10 (9.0%)	
Endometrial thickness, mm		5.62 ± 2.11	6.33 ± 2.29	0.004*
Uterine volume, cm^3^		42.59 ± 15.73	45.82 ± 18.05	0.083
Preoperative AFS		8 (7–10)	8 (6–9)	0.006*
Degree of adhesion	Moderate	161 (61.2%)	82 (73.9%)	0.019*
	Severe	102 (38.8%)	29 (26.1%)	
Adhesion type	Mixed type	139 (52.9%)	52 (46.8%)	0.289
	Peripheral type	124 (47.1%)	59 (53.2%)	
IU depth preop, cm		7.44 ± 0.85	7.52 ± 0.77	0.383
Uterine horn closure preop, *n*	0	168 (63.9%)	80 (72.1%)	0.056
	1	40 (15.2%)	20 (18.0%)	
	2	55 (20.9%)	11 (9.9%)	
Tubal ostia preop	0	82 (31.2%)	20 (18.0%)	0.017*
	1	49 (18.6%)	23 (20.7%)	
	2	132 (50.2%)	68 (61.3%)	
Isolation barrier	Foley balloon	72 (27.4%)	24 (21.6%)	0.021*
	IU suitable balloon	46 (17.5%)	10 (9.0%)	
	IUD	145 (55.1%)	77 (69.4%)	
Menstrual patterns postop	No improvement	46 (17.5%)	4 (3.6%)	0.001*
	Improvement	161 (61.2%)	73 (65.8%)	
	Normal	56 (21.3%)	34 (30.6%)	
IU depth postop, cm		7.34 ± 0.85	7.65 ± 0.68	0.001*
Tubal ostia postop	0	49 (18.6%)	6 (5.4%)	< 0.001*
	1	46 (17.5%)	13 (11.7%)	
	2	168 (63.9%)	92 (82.9%)	
Postop AFS		0 (0–5)	0 (0–0)	< 0.001*

*Preop* preoperative, *TCRA* transcervical resection of adhesions

After the operation, 57 patients tried in vitro fertilization; among whom 16 became pregnant (28.1%, 16/57) and there were 13 full-term live births (22.8%, 13/57). Among 317 women who tried natural conception, 136 became pregnant (42.9%, 136/317), and there were 98 women with live births (30.9%, 98/317).

Among the live births, 57 women experienced cesarean section. There were 10 cases of abnormal placentation, comprising 7, 2, and 1 case of placenta previa, placental implantation, and placental adhesion, respectively. Three women underwent premature delivery, with one case each of placenta previa bleeding cesarean section, premature rupture of membranes at 34 weeks of gestation, and cervical insufficiency at 35 weeks of gestation. There were 41 cases of early pregnancy loss, including 2 of cervical dysfunction and 3 of ectopic pregnancy (1 case of cervical pregnancy).

During the second look 3 months after surgery, 119 patients had various degrees of adhesion, as follows. Among 23 women with mild re-adhesion, 8 were pregnant, and 6 had given live birth (26.1%). Among 83 patients with moderate re-adhesion, 22 were pregnant, and 15 had given live birth (18.1%). There were 13 patients with severe re-adhesion, and none had become pregnant.

### Decision tree model of live birth after TCRA

Based on the ranking results of the average accuracy reduction index (Table 3) with the training set samples, the top 10 variables were used to build the decision tree. In this decision tree the following were classified as node variables for obtaining live birth after IUA surgery: postoperative menstruation; uterine operation times; postoperative uterine cavity depth; postoperative AFS score; and uterine volume. The postoperative menstrual status was the root node variable; uterine volume and postoperative menstrual status appear twice in the model.

**Table 3:** Average accuracy reduction score

	Variables	MDA	MDG
1	IU depth postoperative	0.088	9.838
2	Endometrial thickness	0.070	9.125
3	Uterine volume	0.051	12.413
4	Times of pregnancy	0.048	7.370
5	Age	0.047	9.503
6	Menstrual patterns postoperative	0.046	8.111
7	IU operations	0.040	7.959
8	Isolation barrier	0.038	5.227
9	Postoperative AFS	0.034	4.773
10	Tubal ostia postoperative	0.031	3.518
11	IU depth preoperative	0.031	6.599
12	Preoperative AFS	0.029	5.385
13	Menstrual pattern	0.029	5.979
14	Uterine horn closure preoperative	0.024	3.671
15	Pregnancies lost	0.023	3.242
16	Adhesion type	0.021	2.202
17	Tubal ostia preoperative	0.021	2.849
18	Etiology	0.012	2.568
19	History of TCRA	0.012	1.698
20	Degree of adhesion	0.008	1.298
21	Chief complaint	0.008	1.697
22	Childbirth	0.007	1.393

Abbreviations: *MDA* mean decrease in accuracy, *MDG* mean decrease in Gini, *TCRA* transcervical resection of adhesions

The postoperative menstrual patterns of women who had normal menstruation prior to surgery were assessed as normal, improved, or not improved. The pregnancy rate of patients without improvement (8.1%) was significantly lower than that of the patients with improved or normal menstruation (8.1% cf. 51.0%). Among the patients with improved menstrual volume after surgery, the live birth rate of those who had undergone a previous uterine cavity operation (54.3%, 152/280) was significantly higher than that of patients without a previous procedure (5%, 1/20).

**Fig. 1:**
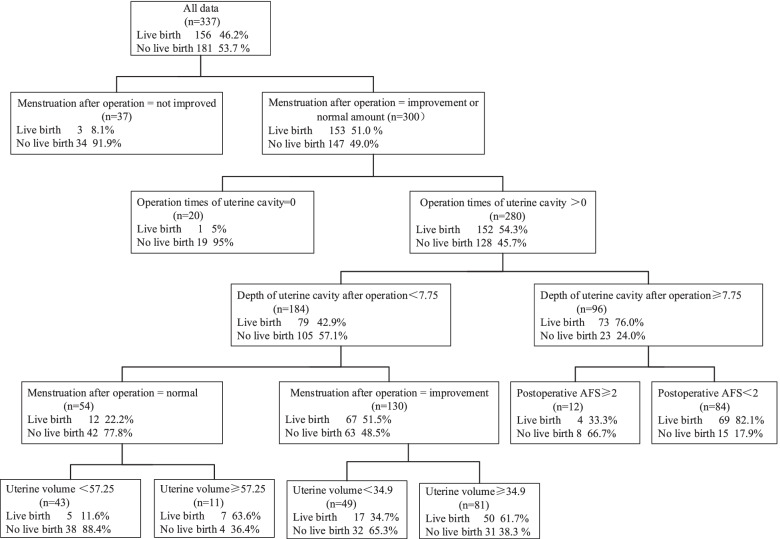
Decision tree model of live birth after TCRA

Among the patients with a previous history of uterine cavity surgery, during hysteroscopic exploration at postoperative 3 months the factor that affected live birth was depth of the uterine cavity. The live birth rate of patients whose depth of uterine cavity was ≥7.75 cm was 76.0% (42.9%). In the patients with a uterine cavity depth < 7.75 cm, those with improved menstruation had a significantly higher live birth rate than did those with normal menstruation. Among patients with improved menstruation, the live birth rate was 61.7% with a uterine volume ≥ 34.9 cm^3^. In patients with normal menstruation, the live birth rate was 63.6% and the uterine volume was ≥57.25 cm^3^. In the subgroup of patients with a uterine cavity depth ≥ 7.75 cm, the live birth rate of those with a postoperative AFS score < 2 was 82.1% (69/85; Fig. 1).

### Performance evaluation of decision tree model in predicting live birth after TCRA

**Fig. 2:**
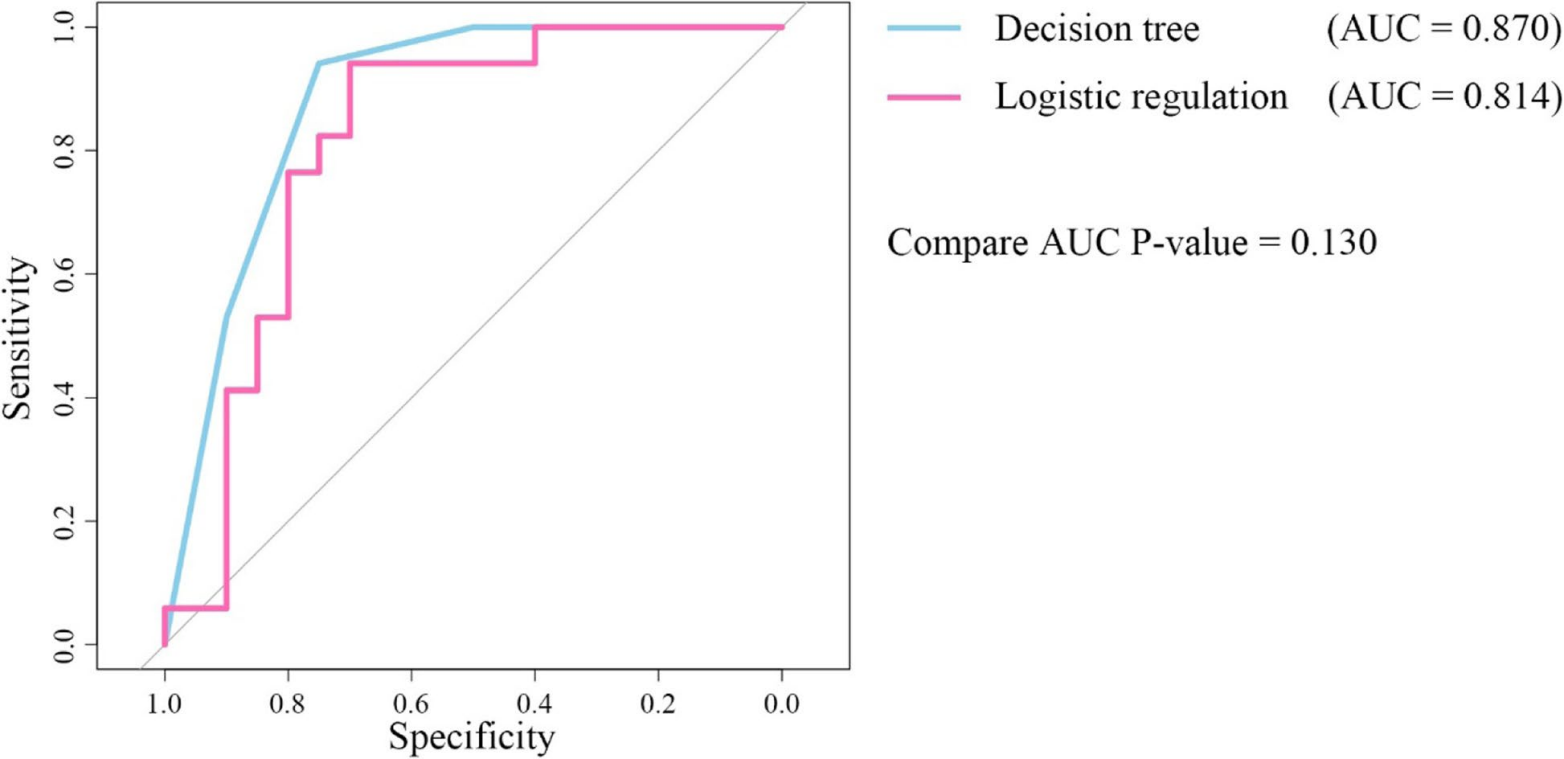
ROC curve comparison between decision tree classification predictive model and logistic regression predictive model (*z* test, *P* = 0.130)

The predictive accuracy of the decision tree model is 83.8% (Table 4; Fig. 2). The sensitivity and specificity were 0.75 and 0.94, respectively. The area under the receiver operating characteristic curve (AUC) is 0.870 (95% confidence interval [CI] 7.699–0.989), and it has high predictive ability. The predictive accuracy of the multivariate logistic regression model is 75.7%, the sensitivity and specificity were 0.76 and 0.75. The AUC is 0.814 (95% CI 0.667–0.962). There is no statistical difference between the two models (*P* = 0.130; *z* test).

**Table 4:** Multivariate logistic regression analysis

	Beta	OR	95% CI of OR	*P*
IU depth postoperative	0.447	1.564	(1.086–2.253)	0.016*
Uterine volume	−0.003	0.997	(0.981–1.013)	0.683
Endometrial thickness	0.180	1.197	(1.054–1.361)	0.006*
IU operations	− 0.078	0.925	(0.691–1.239)	0.602
Age	0.017	1.017	(0.954–1.084)	0.606
Menstrual patterns postoperative	0.018	1.018	(0.671–1.781)	0.934
Times of pregnancy	0.321	1.379	(1.067–1.781)	0.014*
Isolation barrier = balloons*	−0.981	0.375	(0.148–0.949)	0.038*
Isolation barrier = IUD	0.028	1.029	(0.591–1.791)	0.920
Tubal ostia postoperative	0.525	1.690	(1.104–2.589)	0.016*
Postoperative AFS	−0.060	0.942	(0.846–1.048)	0.274

Note: *P* < 0.05 is statistically significant

*Balloons consist of Foley balloon and IU suitable balloon

## Discussion

This study is the first to establish and verify a decision tree model of postoperative live birth in patients with IUAs. The accuracy of the model is 83.8%, which is similar to the accuracy of the multifactor logistic regression analysis model, and has a high predictive value. The structure of the decision tree model clearly depicts the decision-making process, the population characteristics, and the probability of live birth. By establishing a decision tree model we can preliminarily understand the probability of live birth of those with relevant characteristics. This facilitates communication between doctors and patients when deciding to further treatment. On the other hand, it was found that factors closely related to live birth, such as change in postoperative menstrual volume, were the main factors to predict live birth. The operator should pay attention to the identification and protection of the functional endometrium when performing uterine surgery. This study found that the preoperative AFS score is not the primary factor affecting live birth. Therefore, we need to establish a diagnostic scoring standard of intrauterine adhesion for predicting live birth in combination with relevant factors in the decision tree.

The decision tree model we developed is valuable for clinical application, as it characterizes the population, with live birth as the outcome index. Firstly, the decision tree model has high predictive efficiency, as the test set data was validated. At the same time, a multifactor logistic regression model was established. The predictive accuracy of the two models is similar, but the decision tree model is easier to implement, understand, and explain [26].

Secondly, the tree structure is easy to build and understand. It provides clinicians with an appropriate reference for clinical decision-making and facilitates communication with patients. The logistic regression model is displayed in the form of equations, which is difficult to apply in clinical practice. In this study, the predictive index is quantified and sequenced in advance [27]. The decision tree model is simplified by feature extraction, which makes it easier to apply.

In addition, the structure of the decision tree model clearly shows the rates of live birth in patients with various characteristics. The clinician can choose whether to operate based on the local medical technology and patient’s situation, by combining the relevant preoperative predictive factors. This avoids blind, excessive, and ineffective treatment, and simultaneously provides consultation for patients. At the same time, patients with other characteristics are classified, which is more instructive and practical than only analyzing which variables affect the outcome [26].

The current decision tree model not only shows the probability of live birth of patients with different characteristics, but also shows the related factors and their relationships. The closer the root node is to the classification variables, the greater the influence on the outcome. The interactions among variables are shown. Using the decision tree model, we found that postoperative menstrual pattern was the main factor affecting the likelihood of live birth. Although surgery can restore the anatomical structure of the uterine cavity and expand its volume, damage of the basement layer leads to endometrial repair obstacles and local fibrosis [28]. The lack of functional endometrium affects the subsequent fertility outcome [4, 29]. Specifically, the action mode of a variable in the subgroup may be analyzed. For example, in patients with a postoperative uterine cavity depth < 7.75 cm, the pregnancy rate of those with normal menstruation will be lower than that of patients with improved menstruation.

Previous studies have shown that AFS score is closely related to treatment outcome [30–32]. In the decision tree, the AFS score before the operation and in the second look is not the main factor to affect live birth. The AFS score is affects live birth in patients with certain characteristics, the live rate of some patients with AFS score less than 2 was significantly increased. The outcome index of the decision tree model is live birth, while previous studies mainly focus on observing pregnancy or readhesion. In addition, the algorithm of the decision tree is based on the principle of local optimum.

The low rates for pregnancy and live births in women with moderate and severe IUAs have always been a major problem for clinicians. In the present study, the total pregnancy and live birth rates in women with moderate and severe adhesions were relatively low. This may be because, first, the focus of our center is severe adhesions, and data for mild adhesions is not included [20].

Secondly, some of the patients in the study experienced re-adhesions after surgery. Although mild and moderate adhesions were treated with dilated uterine pressure and blunt lens separation at 3 months after surgery, the outcomes of most of these patients are unknown, due to lack of further hysteroscopic examination. Other patients still had moderate and severe IUAs during the second exploration, but no further operation for separation of adhesions was conducted. Some patients with moderate and severe adhesions will refuse further surgery after improvement of menstruation. The patients included in the research results of Xu et al. [33] were confirmed by hysteroscopy to have no adhesions, and the pregnancy and live birth rates were relatively high.

Early assisted reproductive technology may improve the live birth outcome. Xu et al. [33] followed 151 cases of moderate and severe IUAs. The pregnancy rate was 71.5% (108/151) and the live birth rate was 53% (80/151). Half of the pregnancies were achieved through assisted reproductive technology. In the current study, only 57 patients underwent assisted reproductive technology.

The patients in our center have mainly moderate or severe adhesions, especially peripheral and mixed-dense adhesions. Bipolar electric resection has been used for many years. During surgery, protection of the residual endometrium results in better rates of pregnancy and live birth. However, considering that the cold knife does little damage to normal endometrium and affects pregnancy outcome [34], we also use the cold knife gradually.

There are some deficiencies in this retrospective study. The sample size is relatively small, and some predictive variables may not be included. Some variables need to be further quantified and standardized. The menstrual volume should be quantified, but based on the present retrospective data, the range of menstrual volume reduction is too broad. The range of patients’ features is relatively broad, although realistic. The decision tree itself has its shortcomings. The selection of parameters, the number of samples, and the algorithm all affect the stability and predictive accuracy of the decision tree [26]. We will conduct a multicenter prospective study to reduce bias and further stabilize the decision tree.

## Conclusion

The decision tree model of live birth after surgery for IUAs can identify the characteristic population and predict the probability of live birth. This model will help clinicians make appropriate clinical decisions during patient consultations.

## Data Availability

The datasets used and/or analysed during the current study are available from the corresponding author on reasonable request.
